# Precision at the Cutting Edge: Ex Vivo Confocal Microscopy for Perioperative Tumour Thickness Assessment in Melanoma

**DOI:** 10.1111/exd.70136

**Published:** 2025-07-01

**Authors:** D. Hartmann, A. Swarlik, L. Buttgereit, L. Stärr, K. Kerl‐French, M. Flaig, E. C. Sattler, L. E. French, M. Deußing

**Affiliations:** ^1^ Department of Dermatology and Allergy LMU University Hospital, LMU Munich Munich Germany; ^2^ Department of Dermatology and Allergy Munich Municipal Hospital Munich Germany; ^3^ Department of Dermatology and Cutaneous Surgery Miller School of Medicine, University of Miami Miami Florida USA

**Keywords:** dermatology, fluorescence microscopy, melanoma, non‐invasive imaging, reflectance microscopy

## Abstract

Ex vivo confocal laser microscopy (EVCM) represents a promising diagnostic tool for the immediate assessment of fresh tissue, with significant potential for the management of melanoma. This study aimed to evaluate the accuracy of EVCM in determining perioperative tumour thickness, a critical factor in guiding treatment strategies for melanoma. A total of 27 confirmed melanomas of varying thickness and from multiple anatomic sites were analysed using both EVCM and gold standard conventional histopathology. Tumour thickness was independently measured using confocal tumour thickness (CTT) and histopathological tumour thickness (HTT) and subsequently compared using correlation analysis, Spearman's correlation coefficient and Bland–Altman plot. Our findings demonstrate a high correlation between HTT and CTT, with a Spearman's correlation coefficient of 0.94. Bland–Altman analysis revealed a mean difference of −0.19 ± 0.72 mm between CTT and HTT, indicating a strong agreement between the two measurement methods. These results underscore the potential of EVCM as a reliable tool for perioperative evaluation of tumour thickness in melanoma, potentially streamlining the decision‐making process for surgical margins and improving patient outcomes. Further studies with larger sample sizes are warranted to validate these findings and explore the broader applicability of EVCM in clinical practice.

## 1 Introduction

Melanoma is one of the most aggressive forms of skin cancer, with a rising incidence worldwide [1]. The WHO counted 331.722 new cases of melanoma worldwide in the year 2022 and 58.667 melanoma‐associated deaths [2, 3]. Accurate assessment of tumour thickness according to Breslow [4] is paramount, as it directly influences treatment decisions, particularly the extent of surgical excision required to prevent recurrence as well as the need for sentinel lymph node biopsy (SNLB) [5]. Traditionally, histopathological evaluation using conventional histopathology has been the gold standard for determining tumour thickness [6]. However, this process can be time‐consuming, often delaying critical perioperative decisions.

Ex vivo confocal laser microscopy (EVCM) has emerged as a novel diagnostic technique with the potential to revolutionise the rapid assessment of fresh tissue [7]. By providing high‐resolution imaging of tissue architecture within minutes after surgical removal, EVCM allows for the immediate evaluation of tumour characteristics, including thickness, without the need for extensive tissue processing [8, 9]. This capability offers significant advantages in the perioperative setting, where timely decision‐making is crucial.

Previous studies have demonstrated the utility of EVCM in the evaluation of various skin conditions, including benign and malignant tumours [10, 11, 12, 13]. However, its application in measuring tumour thickness in clinical practice remains under investigation. In 2016, our team conducted a pilot study, examining the therapeutic accuracy of the confocal measured tumour thickness using EVCM in black and white fluorescence and reflectance mode [14]. Over the last years, the technology evolved and thus offers new technical and software features. Nowadays, new generation EVCM devices provide an additional mode to generate a coloured digitally stained HE‐like image, which makes the image evaluation easier, especially for pathologists, who are more familiar with conventional histopathological haematoxylin and eosin (HE) slides [15]. Given the pressing need to update and validate our pilot study findings, this study aimed to assess the concordance between the confocal tumour thickness (CTT) measured in digital HE (DHE) EVCM images and histopathological tumour thickness (HTT) on traditional HE slides in a larger cohort of confirmed melanoma cases. By analysing the correlation between these two methods and exploring the potential of EVCM for immediate perioperative decision‐making, we seek to establish the role of EVCM as a valuable tool in the clinical management of melanoma.

## 2 Materials and Methods

From March 2023 until June 2024, patients with a suspected diagnosis of melanoma of the skin were recruited at the Department of Dermatology and Allergy, University Hospital, LMU Munich, Munich, Germany. All patients gave written consent to participate in this study, approved by the local ethics committee of LMU Munich (Ref.‐Nr. 19‐150 and Ref.‐Nr. 23‐0393).

Suspected tumours were excised from various anatomical sites during routine surgical procedures. Immediately after excision, each tumour specimen was covered with saline solution and transferred for EVCM imaging. The sample was immediately stained according to a previously established protocol [10]. For 30 s each, the tissue was immersed in ethanol (0.7 mmol/L), acridine orange (AO) (0.04 mmol/L; Sigma‐Aldrich, St. Louis, MO, USA), FCF Fast Green (0.067 mmol/L; Sigma‐Aldrich, St. Louis, MO, USA) and NaCl (0.09 mmol/L). Then, the fresh tissue specimens were placed with the cut surface on an object slide and fixated with sponges and magnets [16, 17].

EVCM imaging was performed using the VivaScope 2500 G‐4 device (VivaScope, Munich, Germany). By combining two lasers with two different wavelengths, being 488 nm (blue, fluorescence) and 638 nm (red, reflection) simultaneously the device creates a digitally stained HE image [15].

The confocal tumour thickness (CTT) was measured directly on the DHE images perpendicular to the epidermis from the *stratum granulare* (granular layer) to the deepest infiltrating tumour cells, according to the established Breslow method [4]. Measurements (CTT) were performed by a blinded and experienced EVCM specialist and skin pathologist (D.H.) using ECVM images in DHE mode.

**FIGURE 1:**
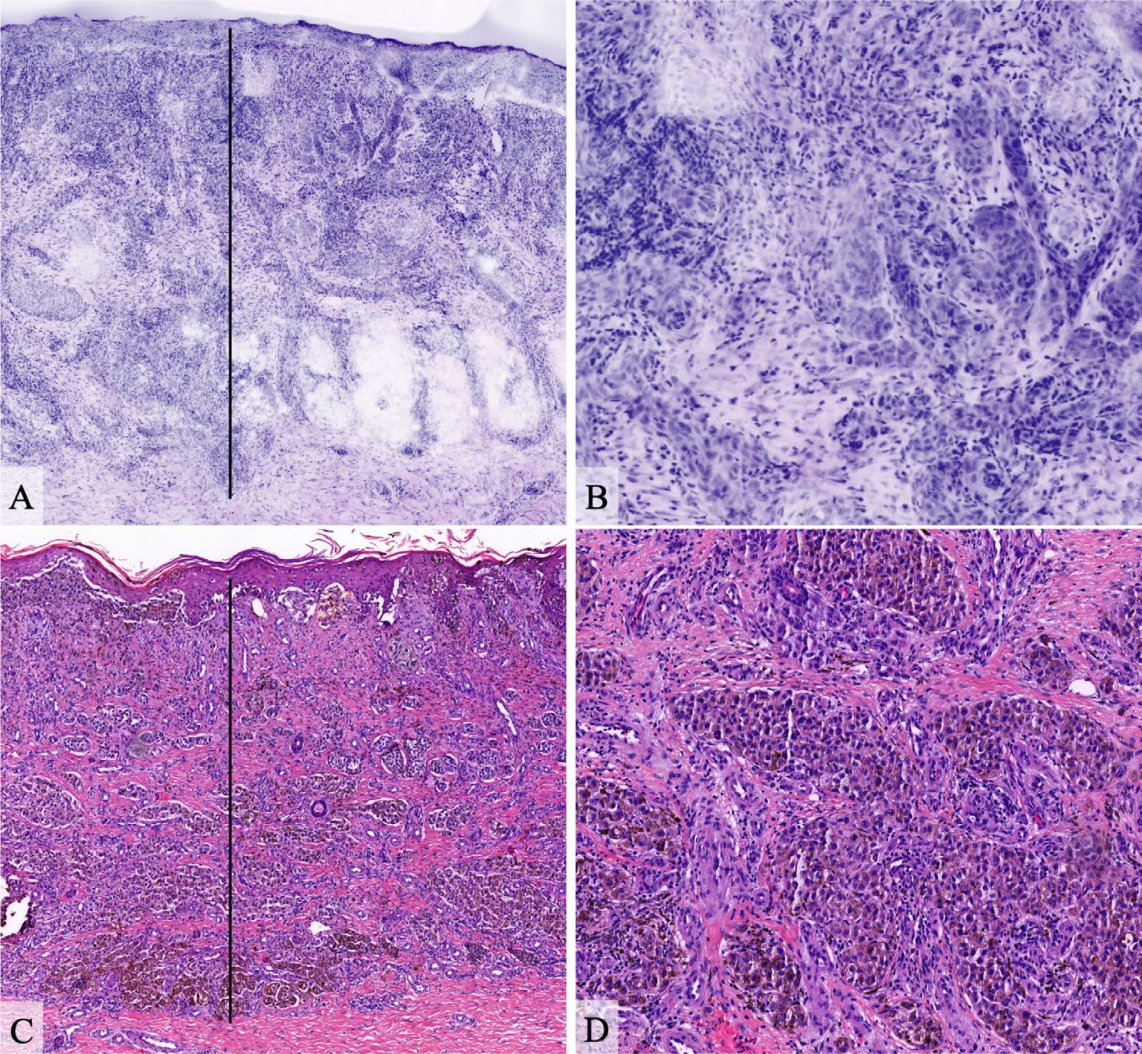
Overview image of a superficial spreading melanoma in digital HE mode using ex vivo confocal laser scanning microscopy (A), detailed view showing irregular and enlarged nests of melanocytes (B). Corresponding conventional histopathology using haematoxylin–eosin staining in overview (C) and detailed view (D). Tumour thickness (2.0 mm) was measured perpendicular to the epidermis from the *stratum granulare* (granular layer) to the deepest infiltrating tumour cells, accordingly to the established Breslow method (black lines).

After the imaging process was completed, the fresh tissue sample was fixed in formalin and underwent standardised routine histopathological examination. Haematoxylin and eosin (HE) staining—and when necessary additional immunohistochemical staining, such as Melan‐A, HMB‐45, S100 and MPM‐2, was performed on the sections, and histopathological tumour thickness (HTT) was measured using the HE slides by an experienced dermatopathologist, blinded to the EVCM results. Analogously to CTT, Breslow thickness was determined as the distance from the granular layer of the epidermis to the deepest point of melanoma invasion (Figure 1) [4].

Both measurements were then compared using statistical methods, including Spearman's correlation coefficient to assess the strength of the relationship between CTT and HTT [18]. Additionally, Bland–Altman plots were generated to evaluate the agreement between the two measurement techniques and to determine any systematic bias [19].

To investigate if fixation in formalin has a possible influence on tumour thickness measurement due to retraction or shrinking artefacts, a subgroup of 10 tissue samples was imaged both native and after fixation in formalin for 24 h. Possible landmarks of the same cellular patterns were measured and compared.

The findings of the formalin effect are presented in Section 3 together with the primary objective of this study.

## 3 Results

In the tumour thickness study, in total 27 tissue samples with histologically confirmed melanoma (15 superficial spreading melanoma, 6 lentigo maligna melanoma, 4 nodular melanoma, 1 acral lentiginous melanoma and 1 cutaneous melanoma metastasis) were included and investigated regarding the measured HTT and CTT. Samples were retrieved from head (*n* = 4), trunk (*n* = 9) and extremities (*n* = 14). Mean age of the patients was 69.2 years, ranging from 36 to 96 years. Further demographic details are provided in Table 1.

**TABLE 1:** Demographic details of investigated patients.

Melanoma subtype	
Superficial spreading melanoma	15
Nodular melanoma	6
Lentigo maligna melanoma	4
Acral lentiginous melanoma	1
Cutaneous melanoma metastasis	1
Age	
Mean age	69.2
Age range	36–96
Gender	
Men	17
Women	10
Donor site	
Head	4
Trunk	9
Extremities	14

**FIGURE 2:**
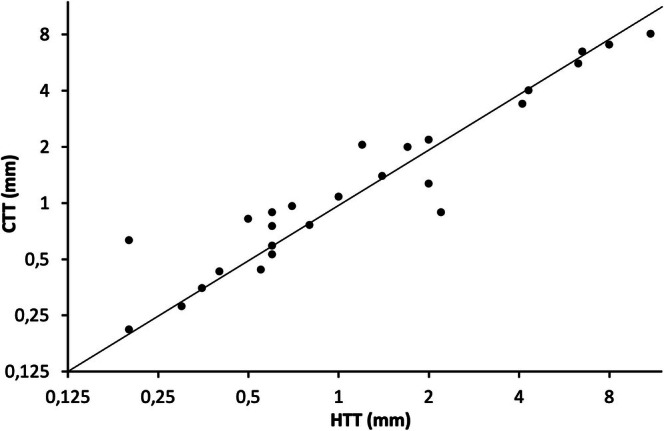
Correlation curve of the histological tumour thickness (HTT) and the confocal tumour thickness (CTT) displayed in logarithmic millimetre scale (mm) showing a strong positive relationship with a Spearman's correlation coefficient of 0.94 (*p* < 0.001).

CTT measurements obtained from EVCM showed a high degree of correlation with HTT measured by conventional histopathology. Spearman's correlation coefficient between CTT and HTT was 0.94 (*p* < 0.001), indicating a strong positive relationship between these two methods (Figure 2).

**FIGURE 3:**
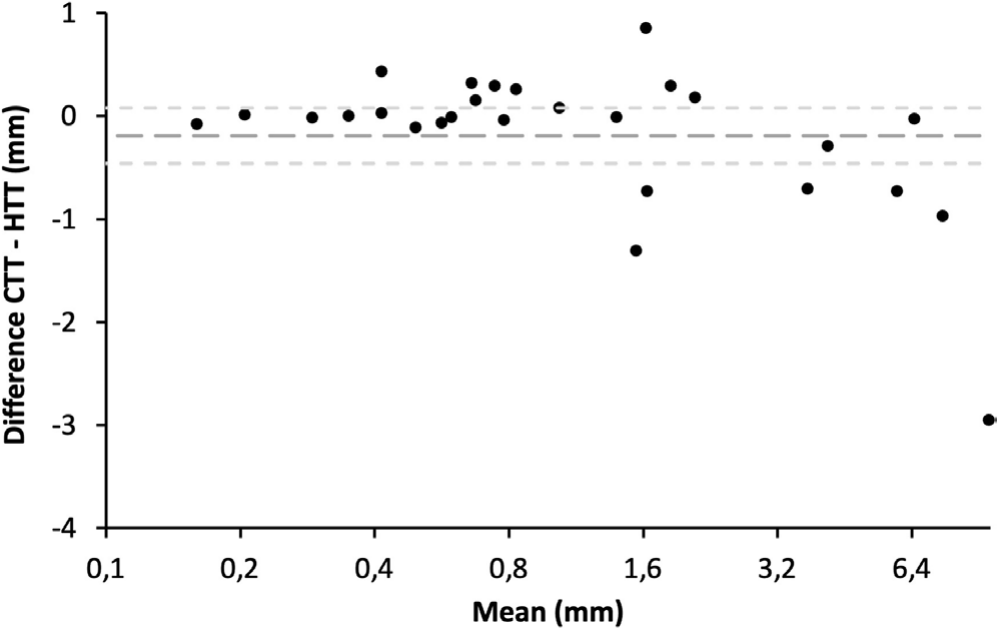
Bland–Altman plot of the measurement of tumour thickness (in mm) by ex vivo confocal microscopy in relation to gold standard histology. The mean difference of −0.19 is presented by the dashed middle line. The 95% confidence interval (CI) for the comparison of CTT to HTT, shown by the outer dashed lines, ranged from −0.46 to 0.08 mm. The differences of each study case are presented by the black dots.

Bland–Altman plots were used to assess the agreement between CTT and HTT. The mean difference between CTT and HTT was −0.19 ± 0.72 mm with a 95% confidence interval (limit of agreement) from −0.46 to 0.08 mm, suggesting that CTT measurements were, on average, slightly lower than HTT measurements. This limit of agreement includes 13 samples and excludes 14 samples, with the highest difference being 2.95 mm and the lowest being 0 mm—the same value as the gold standard. No significant systematic bias was observed, as the differences appeared to be randomly distributed around the mean (Figure 3).

Further analysis of the data revealed that the accuracy of CTT compared to HTT was consistent across different ranges of tumour thickness. Both thin (< 1 mm) and thick (> 4 mm) melanomas showed strong agreement between the two methods, with minimal deviations observed.

**FIGURE 4:**
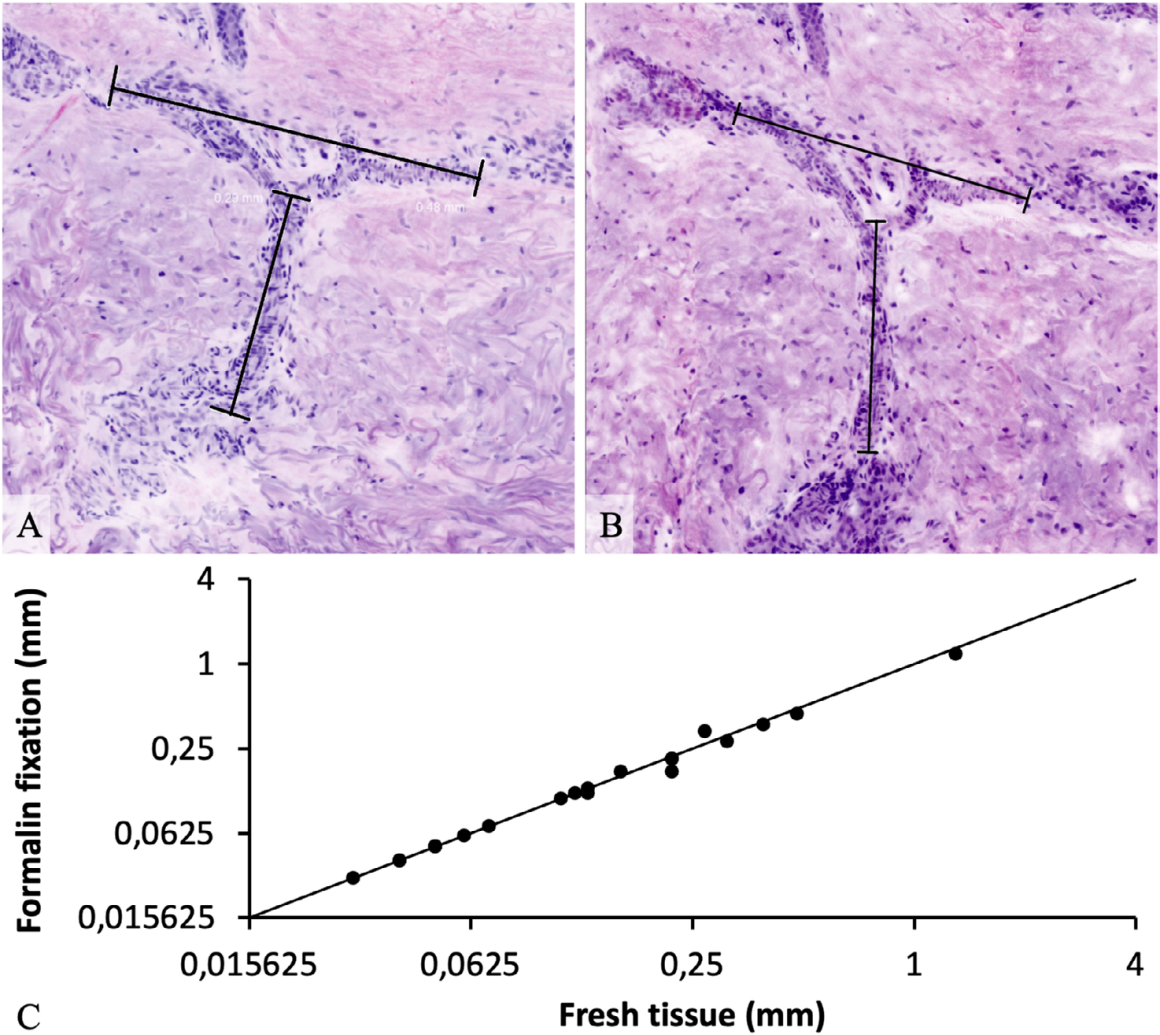
Ex vivo confocal microscopy‐ (EVCM‐) measurement of characteristic cellular patterns/landmarks before (‘fresh tissue’) (A) and after (‘formalin fixation’) (B) incubation in formalin for 24 h. (C) Correlation curve of both measurements displayed in logarithmic millimetre scale (mm) showing strong positive relationship with a Spearman's correlation coefficient of 1 (*p* < 0.001).

For our subgroup analysis, whether fixation in formalin has a possible impact on tissue retraction or shrinkage, 10 skin samples were investigated twice, before and after incubation in formalin. After 24 h fixation, the sample was dyed and imaged again. Those two EVCM pictures were labelled ‘fresh tissue’ and ‘formalin fixation’ and evaluated on possible landmarks of the same cellular pattern. Both images showed a high conformity with a calculated Spearman's correlation coefficient of 1, showing a very strong correlation between the two variables (Figure 4).

## 4 Discussion

In 2016, our working group conducted a pilot study investigating the feasibility of tumour thickness measurement with confocal laser scanning microscopy in relation to the gold standard traditional histopathology [14]. Ten samples of histologically confirmed melanoma were acquired, whereas one sample was excluded due to technical reasons. The confocal measurements were performed by blinded examiners using images in reflectance and fluorescence mode. Since the technology has evolved, we conducted a follow‐up trial with a total of 27 confirmed melanomas using the new generation VivaScope 2500 G‐4 with the possibility of generating a digitally stained HE image to validate and apply our previous findings [14].

Our study confirmed the high accuracy and reliability of EVCM in measuring tumour thickness in melanoma, as compared to conventional histopathology. The strong correlation between CTT and HTT, as indicated by a Spearman's correlation coefficient of 0.94, highlights the potential of EVCM to serve as a valuable tool in the perioperative management of melanoma.

The slight mean difference of −0.19 mm between CTT and HTT, observed in the Bland–Altman analysis, suggests that EVCM tends to slightly underestimate tumour thickness compared to histopathology.

Since we presumed differences in tumour thickness measurement due to fixation in formalin compared to fresh tissue examination, a subset of tissue probes was investigated twice—before and after incubation in formalin. In these cases, we did not see any significant impact on cellular or structural measurement. These findings align with a study conducted by Dauendorffer et al. [20], who examined a possible shrinkage induced by formalin. In this study, the lesion was measured prior to the excision (in vivo), shortly after excision (ex vivo) and after formalin fixation (in vitro). While the measurements showed a shrinkage of 16% in length and 18% in width before and after excision, there was no significant difference between the measurements before and after fixation in formalin.

In our investigation, the main source of error was the cytomorphologic discriminability between inflammatory infiltrates and atypical melanocytic nests using EVCM. Since lymphocytic infiltrates with admixture of plasma cells often surround melanoma lesions [21], the examiner experienced difficulties in differentiating between a deeper tumour infiltration or adjacent inflammation. Especially in one case with a strong inflammatory infiltrate, the examiner deviated strongly in measuring the EVCM tumour thickness from CTT measured in conventional histopathology. In this case, multiple additional sections and immunohistochemical staining, including Melan A and SOX10, were performed, indicating the complexity of the case. While in such cases, additional immunohistochemistry can aid in traditional histopathology, the lack of suitable immunohistochemistry in EVCM poses a disadvantage. Additionally, melanin‐containing cells, such as melanocytes, melanoma cells and melanophages, appear pink when depicted in DHE due to a higher refractive index of melanin. This causes them to deviate from the typical brown appearance seen with conventional HE [9, 22]. While melanin‐containing cells are identified by their pink appearance, distinguishing between a melanocyte and melanoma cell in EVCM can be challenging—leading to possible inaccuracies in tumour thickness assessment using EVCM in cases of melanoma developing on a benign nevus. Therefore, the possibility of using artificial intelligence (AI) based pattern recognition, especially on the subcellular and cellular level, may aid the examiner by analysing the single cells with higher accuracy in the future.

One of the key advantages of EVCM is its ability to provide immediate results within minutes and imaging of fresh tissue without the need for time‐consuming fixation or sectioning. This characteristic makes EVCM particularly useful in the intraoperative setting, where timely information is critical for guiding surgical decisions, such as determining appropriate safety margins or the need for SNLB. Furthermore, EVCM could have a significant impact in regions with limited or no access to diagnostic methods, particularly histology. The potential integration of EVCM allows the clinical team to provide appropriate care in a single visit by using one tool for the entire first‐line therapy. This would make the management of melanoma more accessible worldwide. To investigate if the differences between CTT and HTT measurements would impact clinical decision‐making, a subgroup analysis was performed. According to the German guideline of care for cutaneous melanoma, the individual therapeutic strategies depend on the stage of cancer [23]. One of the most important parameters in this decision constitutes the measured Breslow tumour thickness. According to the mentioned German guidelines, the recommendation for surgical excision of safety margins in melanoma with a tumour thickness under 2.01 mm is 1 cm starting from the clinical edge of the lesion; for a tumour thickness of 2.01 mm or above, it is 2 cm [23]. Twenty‐four out of 27 (=89%) investigated melanomas would have been in the correct surgical safety margin recommendation category whether measured in EVCM or in gold standard histopathology. Two patients would have undergone an ‘overtherapy’ with a broader excision safety margin than necessary, and only one patient would have undergone an ‘undertherapy’ with the actual need for one additional centimetre of safety margin.

The necessity of an additional SLNB is also determined mainly by the Breslow tumour thickness, whereas other possible criteria may influence the decision for a SLNB based on country‐specific guidelines. In our study, the German guidelines were applied: with a tumour thickness above 1 mm, SLNB surgery is recommended to the patients. From 0.75 to 1 mm tumour thickness, the SLNB is considered if additional risk factors are present, such as, that is, a mitosis rate of > 1 mitosis per mm^2^ or patient age below 40 years [23].

For research purposes our working group solely focused on the measurement of the tumour thickness. Further studies are necessary to investigate if an assessment of the mitosis rate is feasible using EVCM. For the artificial scenario in this study design, we applied the condition that all patients present additional risk factors. Out of the 27 investigated samples, 25 (=93%) would have been in the correct SLNB category, whereas the other two patients would have undergone a SLNB, which would not have been necessary according to the above‐mentioned national guidelines.

The agreement between CTT and HTT across different tumour thickness ranges reinforces the potential of EVCM to be used across a broad spectrum of melanoma cases, from thin melanomas to thicker lesions that may necessitate wider excisions and SLNB. With the possibility of acquiring a histological image during the surgery, the treating team would be able to minimise the timespan of the primary excision surgeries, even combining several steps within one surgery. Furthermore, it may even be possible to integrate the diagnosis confirming surgery and the safety margin surgery into one operation by examining the tumour entity and simultaneously measuring the Breslow tumour thickness in order to excise the appropriate safety margin instantly and at one time only. It is important to point out that immediate safety margin excision should only be performed in case no sentinel lymph node biopsy is planned (i.e., < 1.0 mm tumour thickness or < 0.75 mm in patients with risk factors). Otherwise, a wide local primary excision could interfere with lymphatic mapping and the identification of the correct lymph node.

The findings of this study align with previous research that has demonstrated the utility of confocal microscopy in dermatology, particularly in distinguishing between benign and malignant lesions and in assessing other skin tumours. However, this study extends the application of EVCM to the specific and crucial task of measuring tumour thickness in melanoma, a factor that directly influences prognosis and treatment strategies.

Despite the promising results, there are limitations to this study that warrant consideration. The sample size, while sufficient to demonstrate a strong correlation between CTT and HTT, is relatively small. Larger studies are needed to validate these findings across a more diverse population and to assess the consistency of EVCM measurements in tumours with complex or heterogeneous structures. Additionally, while EVCM provides rapid assessments, it requires specialised equipment and expertise, which may limit its widespread availability in broad clinical settings.

Future research should focus on the integration of EVCM into routine clinical workflows and explore its potential to reduce the need for reoperations by providing more accurate initial excisions. Investigating the cost‐effectiveness of EVCM, as well as its application in other types of skin cancers or even non‐dermatological tumours, could further establish its role in modern surgical oncology.

## 5 Conclusion

This study highlights EVCM's ability to deliver immediate, high‐resolution imaging of fresh tissue for intraoperative measurement of tumour thickness in melanoma. The strong correlation between CTT and HTT, with a Spearman's correlation coefficient of 0.94, demonstrates that EVCM can provide reliable real‐time assessments that closely align with traditional histopathological evaluations. While our study supports the integration of EVCM into clinical practice, further research with larger cohorts is needed to confirm these findings and to explore its broader applicability. Additionally, evaluating the cost‐effectiveness and logistical considerations of incorporating EVCM into routine melanoma surgery is pivotal for its widespread adoption.

## Author Contributions

D.H. and M.D. conceived and designed the project. D.H., A.S., L.B., L.S. and M.D. acquired the data. D.H., A.S. and M.D. analysed and interpreted the data. K.K.‐F. and M.F. did the histopathological analysis. D.H., L.E.F. and M.D. did supervision of the study. D.H., A.S. and M.D. wrote the paper. D.H., A.S., E.C.S., L.E.F. and M.D. did revision of the paper.

## Conflicts of Interest

The authors declare no conflicts of interest.

## Data Availability

The data that support the findings of this study are available on request from the corresponding author. The data are not publicly available due to privacy or ethical restrictions.

## References

[1] G. V. Long , S. M. Swetter , A. M. Menzies , J. E. Gershenwald , and R. A. Scolyer , “Cutaneous Melanoma,” Lancet 402, no. 10400 (2023): 485–502, 10.1016/s0140-6736(23)00821-8. 37499671

[2] S. Ugurel and R. Gutzmer , “Melanom,” Journal der Deutschen Dermatologischen Gesellschaft 21, no. 4 (2023): 343–347, 10.1111/ddg.15053. 36999586

[3] J. E. M. Ferlay , F. Lam , M. Laversanne , et al., Global Cancer Observatory: Cancer Today (International Agency for Research on Cancer, 2024), https://gcoiarcwhoint/today.

[4] A. Breslow , “Thickness, Cross‐Sectional Areas and Depth of Invasion in the Prognosis of Cutaneous Melanoma,” Annals of Surgery 172, no. 5 (1970): 902–908, 10.1097/00000658-197011000-00017. 5477666 PMC1397358

[5] D. Schadendorf , A. C. J. van Akkooi , C. Berking , et al., “Melanoma,” Lancet 392, no. 10151 (2018): 971–984, 10.1016/s0140-6736(18)31559-9. 30238891

[6] K. Lallas , A. Kyrgidis , A. Chrysostomidis , E. Vakirlis , Z. Apalla , and A. Lallas , “Clinical, Dermatoscopic, Histological and Molecular Predictive Factors of Distant Melanoma Metastasis: A Systematic Review and Meta‐Analysis,” Critical Reviews in Oncology/Hematology 202 (2024): 104458, 10.1016/j.critrevonc.2024.104458. 39074631

[7] E. Cinotti , J. L. Perrot , B. Labeille , F. Cambazard , and P. Rubegni , “Ex Vivo Confocal Microscopy: An Emerging Technique in Dermatology,” Dermatology Practical & Conceptual 8, no. 2 (2018): 109–119, 10.5826/dpc.0802a08. 29785327 PMC5955077

[8] E. Cinotti , M. Haouas , D. Grivet , and J. L. Perrot , “In Vivo and Ex Vivo Confocal Microscopy for the Management of a Melanoma of the Eyelid Margin,” Dermatologic Surgery 41, no. 12 (2015): 1437–1440, 10.1097/dss.0000000000000517. 26561956

[9] D. Hartmann , C. Ruini , L. Mathemeier , et al., “Identification of Ex‐Vivo Confocal Laser Scanning Microscopic Features of Melanocytic Lesions and Their Histological Correlates,” Journal of Biophotonics 10, no. 1 (2017): 128–142, 10.1002/jbio.201500335. 27091702

[10] D. Hartmann , L. Buttgereit , L. Stärr , E. Sattler , L. French , and M. Deussing , “Intraoperative PRO Score Assessment of Actinic Keratosis With FCF Fast Green‐Enhanced Ex Vivo Confocal Microscopy,” Applied Sciences 14 (2024): 1150, 10.3390/app14031150.

[11] L. Messner , M. Deußing , M. Maurer , et al., “Ex Vivo Confocal Laser Scanning Microscopy in Rare Skin Diseases,” Cancers (Basel) 16, no. 9 (2024): 1713, 10.3390/cancers16091713. 38730676 PMC11083278

[12] G. Vladimirova , C. Ruini , F. Kapp , et al., “Ex Vivo Confocal Laser Scanning Microscopy: A Diagnostic Technique for Easy Real‐Time Evaluation of Benign and Malignant Skin Tumours,” Journal of Biophotonics 15, no. 6 (2022): e202100372, 10.1002/jbio.202100372. 35233962

[13] D. Hartmann , S. Krammer , M. R. Bachmann , et al., “Ex Vivo Confocal Microscopy Features of Cutaneous Squamous Cell Carcinoma,” Journal of Biophotonics 11, no. 4 (2018): e201700318, 10.1002/jbio.201700318. 29227042

[14] D. Hartmann , S. Krammer , C. Ruini , T. Ruzicka , and T. von Braunmühl , “Correlation of Histological and Ex‐Vivo Confocal Tumor Thickness in Malignant Melanoma,” Lasers in Medical Science 31, no. 5 (2016): 921–927, 10.1007/s10103-016-1936-5. 27056706

[15] S. Razi , S. Ouellette , S. Khan , K. S. Oh , T. M. Truong , and B. K. Rao , “Role of VivaScope 2500 Ex Vivo Confocal Microscopy in Skin Pathology: Advantages, Limitations, and Future Prospects,” Skin Research and Technology 29, no. 6 (2023): e13388, 10.1111/srt.13388. 37357649 PMC10250963

[16] J. Pérez‐Anker , S. Puig , and J. Malvehy , “A Fast and Effective Option for Tissue Flattening: Optimizing Time and Efficacy in Ex Vivo Confocal Microscopy,” Journal of the American Academy of Dermatology 82, no. 5 (2020): e157–e158, 10.1016/j.jaad.2019.06.041. 31255748

[17] J. Pérez‐Anker , A. Toll , S. Puig , and J. Malvehy , “Six Steps to Reach Optimal Scanning in Ex Vivo Confocal Microscopy,” Journal of the American Academy of Dermatology 86, no. 1 (2022): 188–189, 10.1016/j.jaad.2021.01.044. 33476729

[18] A. Hazra and N. Gogtay , “Biostatistics Series Module 6: Correlation and Linear Regression,” Indian Journal of Dermatology 61 (2016): 593–601, 10.4103/0019-5154.193662. 27904175 PMC5122272

[19] O. Gerke , “Reporting Standards for a Bland‐Altman Agreement Analysis: A Review of Methodological Reviews,” Diagnostics (Basel, Switzerland) 10, no. 5 (2020): 334, 10.3390/diagnostics10050334. 32456091 PMC7278016

[20] J. N. Dauendorffer , S. Bastuji‐Garin , S. Guéro , N. Brousse , and S. Fraitag , “Shrinkage of Skin Excision Specimens: Formalin Fixation Is Not the Culprit,” British Journal of Dermatology 160, no. 4 (2009): 810–814, 10.1111/j.1365-2133.2008.08994.x. 19183182

[21] W. Kempf , M. Hantschke , and H. Kutzner , Dermatopathologie, 4th ed. (Springer, 2020), 10.1007/978-3-662-59240-3.

[22] M. Schüürmann , M. M. Stecher , U. Paasch , J. C. Simon , and S. Grunewald , “Evaluation of Digital Staining for Ex Vivo Confocal Laser Scanning Microscopy,” Journal of the European Academy of Dermatology and Venereology 34, no. 7 (2020): 1496–1499, 10.1111/jdv.16085. 31732988

[23] Leitlinienprogramm Onkologie (Deutsche Krebsgesellschaft DK, AWMF) , “Diagnostik, Therapie und Nachsorge des Melanoms, Langversion 3.3,” 2020, AWMF Registernummer: 032/024OL, http://www.leitlinienprogrammOnkologiede/Leitlinien/Melanom/.

